# High conductivity graphite paste for radio frequency identification tag with wireless hydrogen sensor based on CeO_2_–Fe_2_O_3_–graphene oxide†

**DOI:** 10.1039/d5ra00587f

**Published:** 2025-04-22

**Authors:** Hossein Mojtabazadeh, Javad Safaei-Ghomi

**Affiliations:** a Department of Organic Chemistry, Faculty of Chemistry, University of Kashan P.O. Box 87317-51167 Kashan I. R. Iran safaei@kashanu.ac.ir +98-31-55552935 +98-31-55912385

## Abstract

Radio frequency identification (RFID) technology has made significant strides in recent years, opening up a world of possibilities for various industries. However, to achieve success, reliable and accurate real-time data is crucial. One exciting application of RFID technology is fast and wireless detection of gases. Hydrogen, in particular, is considered a clean fuel. However, it is highly flammable, and detecting it quickly and accurately is challenging in various industries. In this regard, our research focuses on developing a high-conductivity graphite paste for RFID tags integrated with a wireless hydrogen sensor based on nano-CeO_2_–Fe_2_O_3_–graphene oxide. In this work, we obtained a graphite paste using Ultra High Power (UHP) graphite electrodes with a high conductivity of 4.75 × 10^5^ S cm^−1^ for non-metallic substrates and 4 × 10^6^ S cm^−1^ with aluminum substrate. Furthermore, we incorporated a hydrogen gas detection sensor into the RFID tag utilizing graphene oxide and cerium oxide–iron oxide nanoparticles. The sensor demonstrated high sensitivity to low concentrations of H_2_ gas (1 ppm), with stable and repeatable performance. The wireless sensing response was evaluated through reflection coefficient (*S*_11_) measurements, confirming effective impedance matching between the RFID chip and antenna. Through this research, we aim to promote the advancement of RFID technology by introducing a low-cost, battery-free sensing platform using graphite and nano-engineered materials, suitable for diverse industrial applications.

## Abstract 2

Battery-free RFID tag using high-conductivity graphite paste and nano-CeO_2_–Fe_2_O_3_–GO sensor enables wireless hydrogen detection with high sensitivity, stability, and long read range for smart sensing applications.

## Graphical abstract

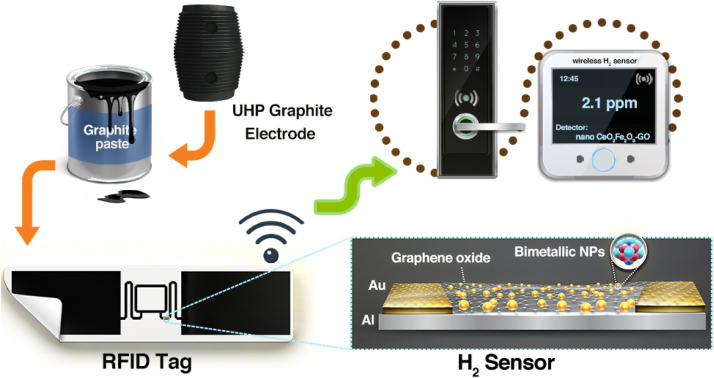

## 1. Introduction

The utilization of wireless sensors in communication networks is a significant approach due to its simple installation process (without the need for wiring) and effortless transfer. Radio Frequency Identification (RFID) is a wireless identification system that enables communication of data between a reader and a tag attached to an object, product, or the like. This smart wireless sensor system utilizes electronic and electromagnetic signals to read and write data without physical contact. Currently, RFID technology has various applications such as access control, barcode enhancement, waste management, telemetry, human implantation, infrastructure management, and security. The use of this technology is increasing in diverse industries.^1–3^ There are two categories of RFID systems available: passive electronic tag: in this type of RFID system, the electronic tags do not require a battery and instead obtain their required energy from the reader unit. The reader unit releases a range of energy up to a few meters to provide the energy needed by the electronic tags in its vicinity. The electronic tag receives the energy transmitted from the card reader, activates and sends its identification information. Active electronic tag: the active RFID system uses electronic tags with an internal power supply to enhance the coverage range. These tags have a battery and emit radio frequency pulses after a set time. Active electronic tags can be read at greater distances (more than 10 meters) than passive electronic tags. However, active electronic tags have some issues, such as their large size (due to the presence of a battery), short lifespan (when the battery runs out, it becomes unusable), high cost per tag, and variable emission rates.^4,5^ The creation of RFID tags involves utilizing appropriate technologies such as screen printing, which is favored for its low cost, minimal pollution, and straightforward preparation. However, the printable inks or pastes utilized in these tags must be both affordable and highly conductive. As a result, various materials have been implemented, including silver pastes,^6,7^ carbon nanotubes,^8^ and graphene.^9–13^ While graphene-based tags are economical, their conductivity is somewhat limited. Researchers have conducted numerous studies aimed at enhancing the electrical conductivity of these RFID tags.

Graphite electrodes made from petroleum coke and needle coke are a source of high conductivity and low resistance graphite. These electrodes can be categorized based on their power rate as regular power, high power, and ultra-high power (UHP). UHP graphite electrode is an ultra-strong graphite-based conductive material that uses petroleum coke and needle coke as aggregates, coal asphalt as a binder, and undergoes complex processes such as calcining, crushing, grinding, batching, mixing, shaping, roasting, impregnation, graphitization, and mechanical processing. It finds application in industries that require ultra-high power steelmaking electric arc furnaces due to its lower resistance, better electrical conductivity, and higher mechanical strength.^14–16^ In this research, we intend to prepare an RFID tag based on UHP graphite electrode with high conductivity and low cost.

Hydrogen (H_2_) is an eco-friendly and unpolluted fuel that does not emit carbon dioxide (CO_2_) when burned. It can be effortlessly generated by electrolyzing water. H_2_ is a highly flammable, odorless and colorless gas, hence detecting H_2_ gas leaks is crucial.^17–19^ Different types of sensors have been created for H_2_ detection, such as thermal conductivity detectors, electrochemical sensors, semiconducting metal oxides (SMOX),^20^ and catalytic combustion sensors.^21^ SMOX sensors including SnO_2_,^22^ ZnO,^23^ Fe_2_O_3_,^24^ TiO_2_,^25^ WO_3_,^26^ In_2_O_3_ (ref. 27) and CeO_2_ (ref. 28) have been developed for hydrogen detection. However, the use of CeO_2_ for H_2_ detection has not been explored much. CeO_2_ has two stable oxidation states (Ce^4+^ and Ce^3+^) that enable oxygen storage and release. It can transform into a non-stoichiometric oxide without damaging its crystal structure when exposed to high temperatures. Its high thermal stability, oxygen storage capacity,^29^ and rich oxygen vacancies make CeO_2_ ideal for gas detection systems. When combined with Fe_2_O_3_, a mixed oxide is formed that has higher reducibility at low temperatures than pure CeO_2_.^30–32^

The research presents the development of a passive UHF-RFID tag with a wireless H_2_ gas detection smart sensor. The developed passive UHF-RFID tag includes a sensing region composed of CeO_2_–Fe_2_O_3_ nanoparticles supported on graphene oxide.

## 2. Result and discussion

### 2.1 Preparation and properties of graphite paste from UHP graphite electrode

**Fig. 1:**
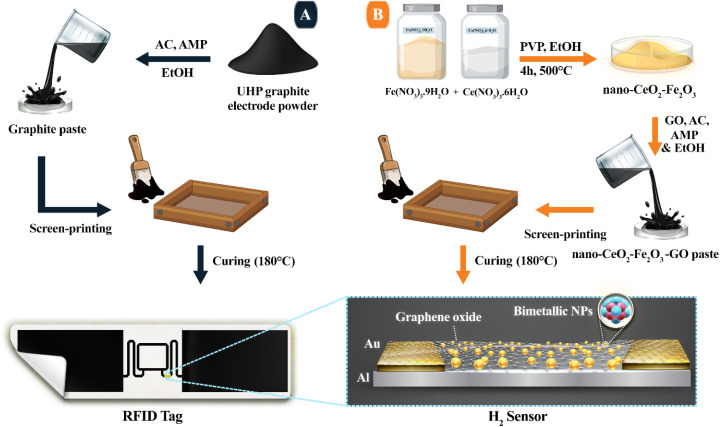
(A) General scheme for graphite paste fabrication and RFID tag screen printing (B) fabrication of nano-CeO_2_–Fe_2_O_3_–GO and chemical resistance H_2_ sensing system with wireless detection.

At first, the UHP graphite electrode (UHP-GE) was completely powdered. Then, a paste was created by adding ethanol, acrylate copolymer (AC), and 2-amino-2-methyl-1-propanol (AMP) (Fig. 1a). The preparation process for the UHP-GE paste is described in detail in the “Experimental” section. The acrylate copolymer was selected due to its excellent adhesion properties and relatively low boiling point 99.5 °C, which allows for decomposition at moderate temperatures. AMP was included as a pH stabilizer and dispersion aid. In comparison with other commonly used binders such as ethyl cellulose, poly(*N*-vinyl-2-pyrrolidone) (PVP), and diblock copolymer containing pendant cholesterol groups, whose decomposition typically requires temperatures above 200 °C,^33,34^ our selected formulation enables efficient curing at 180 °C while maintaining both conductivity and mechanical integrity. Ethanol acts as both a solvent and a dispersant for the UHP-GE powder, allowing it to be well dispersed in the organic phase matrix consisting of AC and AMP. The UHP-GE paste was then homogeneously screen printed onto various substrates, including aluminum foil, fire-resistant paper, and Kapton foil, with a thickness of 25 µm. The screened UHP-GE pastes were cured on different substrates, with the best temperature being 180 °C for 20 minutes (Table S1†). This curing condition was optimized to achieve the best balance between adhesion and electrical conductivity. During the curing process, voids were created, but these were eliminated by pressing the paste. As a result of this process, the graphite adheres to the desired surface, and due to the application of heat, the decomposed AC, ethanol and AMP evaporate. To stick graphite onto a desired surface, additives such as resin, polymer, *etc.* must be used, but these additives increase resistance.

In this research, it was attempted to achieve suitable adhesion of the UHP-GE paste to the surface without negatively affecting resistance by decomposing the AC and removing the solvent and AMP at a temperature of 180 °C.

**Fig. 2:**
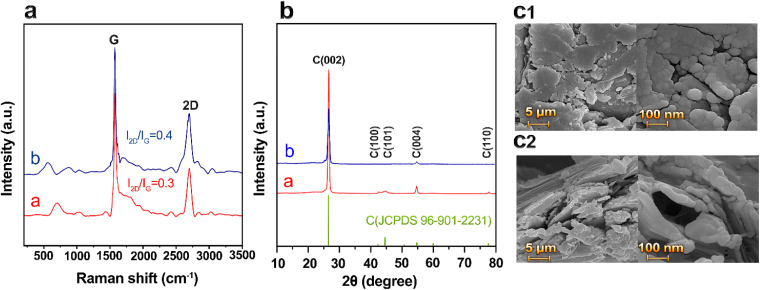
Effects of treatment on UHP-GE. (a) Raman spectrum of UHP-GE (a), Raman spectrum of UHP-GE paste after treatment and curing at 180 °C (b). (b) XRD patterns of UHP-GE before (a) and after (b) treatment. (c1) FESEM images of UHP-GE before treatment and (c2) after.

Analysis of the Raman spectrum demonstrated indications of both loss of solvent and additives, as depicted in Fig. 2a. The sharp peak at approximately 1570 cm^−1^ (G) corresponds to the vibrational mode in the plane that includes sp^2^ hybridized carbon atoms generating the graphite sheets. The band that emerges around 2700 cm^−1^ (2D) is an outcome of the double-phonon lattice vibrational process. The number of graphene layers or stacking can be estimated by the intensity ratio of the 2D and G bands. The higher the ratio, the fewer the number of layers. For instance, monolayer graphene has an *I*_2D_/*I*_G_ ratio of 2.^35^ In sample “a” (UHP-GE), the *I*_2D_/*I*_G_ ratio was 0.3, while in sample “b” (after treatment with additives and solvents and curing at 180 °C), it was 0.4.

X-ray diffraction (XRD) investigation (Fig. 2b) of UHP-GE “a” shows a sharp peak at 26.6°, corresponding to (002). After treatment with additives, solvent, and curing at 180 °C, sample “b” shows a (002) peak with lower intensity compared to “a”. The XRD results were consistent with the graphite standard (JCPDS 96-901-2231). The reduced intensity of the (002) XRD peak in sample “b”, along with the slightly increased *I*_2D_/*I*_G_ ratio in Raman analysis, suggests a mild expansion of interlayer spacing or partial exfoliation of graphite sheets after curing.

Furthermore, the similarity between the spectra and the absence of peaks corresponding to functional groups in the Raman spectrum indicate that AC, ethanol, and AMP were decomposed during the curing process. Fig. 2c1 and c2 are field emission scanning electron microscopy (FESEM) images of samples “a” and “b”. EDS and EDS-MAP (ESI Fig. S1 and S2†) also support the decomposition of AC and evaporation of AMP and solvent components.

**Fig. 3:**
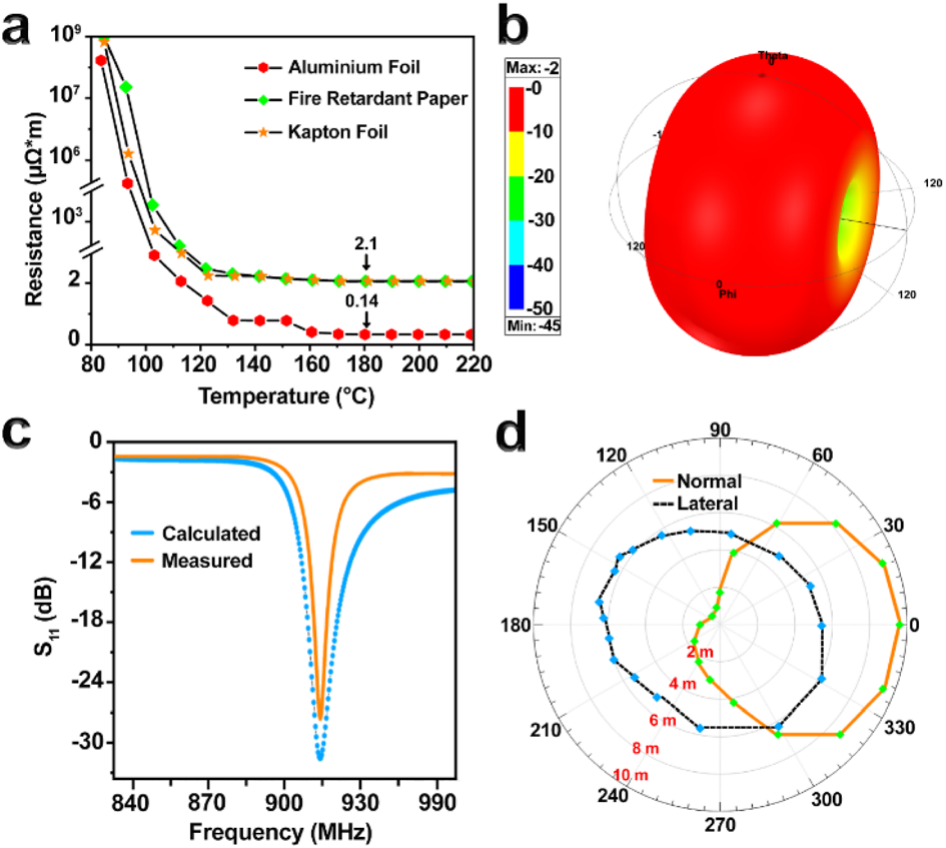
(a) The graph displays the resistance values at different temperatures. (b) The three-dimensional radiation pattern of the UHF dipole RFID tag antenna model was calculated using HFSS. (c) The *S*_11_ results of the fabricated UHF dipole tag antennae were both calculated and measured. (d) The 915 MHz read range pattern of the antenna with an aluminum substrate was recorded, showing superior performance compared to non-metallic substrates (as depicted in ESI Table S3†).

The electrical properties of the prepared films were studied, and it was found that the AC significantly increases the resistance. The initial resistance of UHP-GE pastes screened on different substrates was high (10^9^ µΩ m). The boiling point of the AC is 99.5 °C. After applying heat to 100 °C, the resistance of the films decreased to about 1000 µΩ m, indicating the decomposition of the AC. At a temperature of 180 °C, the resistance reached 0.14 µΩ m for the film with an aluminum substrate and 2.1 µΩ m for fire-resistant paper and Kapton foil (Fig. 3a). The initial resistance of UHP-GE powder was 3.5 µΩ m. After paste formulation and curing, the resistance decreased to 0.14 µΩ m (on aluminum), benefiting from both improved conductivity and strong adhesion. Increasing the graphite layer thickness from 25 µm to 35 µm *via* repeated screen printing increased the resistance to 2.1 µΩ m, indicating that the conductive effect of the aluminum substrate diminishes as the graphite layer becomes thicker. It is clear that in the samples with Kapton foil substrate and fire-resistant paper, the resistance also decreases with the increase in the number of screen printing.

### 2.2 Preparation and properties of UHF RFID tags

In the first stage, the antenna was designed and simulated using Ansys HFSS software. The optimized design was selected based on impedance matching and reduced tag dimensions (Fig. S3†). As explained in the previous section, UHP-GE paste was screen-printed onto the desired surface and heated at 180 °C for 20 minutes. Then, the primary tag, consisting of two layers of aluminum (fire-resistant paper or Kapton foil) and graphite, was glued onto the PET surface. Additionally, it is worth noting that the sheet resistance (*R*_s_) for tags with an aluminum substrate was recorded as 0.01 Ω sq^−1^, while it was 0.08 Ω sq^−1^ for non-metallic substrates (as shown in Table S3†). In the next step, a packaged RFID chip (SL3S1213FTB0) was used, which was glued to the location using silver epoxy resin.

The maximum gain of antennas with non-metallic substrates (fire-resistant paper and Kapton foil) was 1 dB, and the maximum gain of the antenna with an aluminum substrate was −2 dB (Fig. 3b). The measured *S*_11_ values showed good agreement with the simulated results at 915 MHz, confirming proper impedance matching. The “reflection coefficient” (*S*_11_) shows the compatibility of the tag antenna with the RFID chip connected to it. The more negative the value of *S*_11_, the higher the efficiency of the antenna. *S*_11_ was −28 dB for the aluminum substrate antenna (Fig. 3c) and −13 dB for the other antennas (Table S2†). These results show that the proposed antennas match the impedance of the chip at the frequency of 915 MHz. The integration of the sensing layer did not significantly shift the resonance frequency, indicating that the antenna performance remained stable post-fabrication.

Additionally, the reading range of tag antennas with Kapton foil, fire-resistant paper, and aluminum substrates was about 6, 5.9 and 10 m, respectively (Fig. 3d).

The results of this study demonstrate that the graphite tags prepared from UHP-GE paste perform exceptionally well when compared to graphene and even metallic tags (Table 1). Moreover, they are cost-effective and possess long read ranges that make them a viable alternative to expensive tags. By utilizing these tags, we can achieve greater efficiency and accuracy in various industries, ranging from logistics to healthcare.

**Table 1:** Summary of important parameters of carbon-based RFID tags

No.	Material	*R*_s_ a (Ω sq^−1^)	*σ* b (S m^−1^)	Read range (m)	Year/ref.
1	Graphene	5	1 × 10^4^	2.6	2016/36
2	Graphene-nanoflakes	3.8	4.3 × 10^4^	4	2016/37
3	Few-layer graphene	3	1 × 10^4^	11	2019/38
4	Graphene	2.8	3.7 × 10^4^	9	2018/39
5	Graphene	1.9	1.39 × 10^4^	5	2016/40
6	Graphene nanoplatelets	0.04	4.2 × 10^5^	N/A	2018/41
7	Graphene	0.02	1.6 × 10^6^	12	2021/42
8	UHP-GE@ FRP^c^ or UHP-GE@ KF^d^	0.08	4.75 × 10^5^	6	This work
9	UHP-GE@ Al^e^	0.01	4 × 10^6^	10	This work
10	Al	0.00289	1.38 × 10^7^	15.2	This work

a *R*_s_ = sheet resistance.

b *σ* = conductivity.

c FRP = fire-resistant paper.

d KF = Kapton foil.

e Al = aluminium.

### 2.3 Preparation and properties of nano-CeO_2_–Fe_2_O_3_–GO

**Fig. 4:**
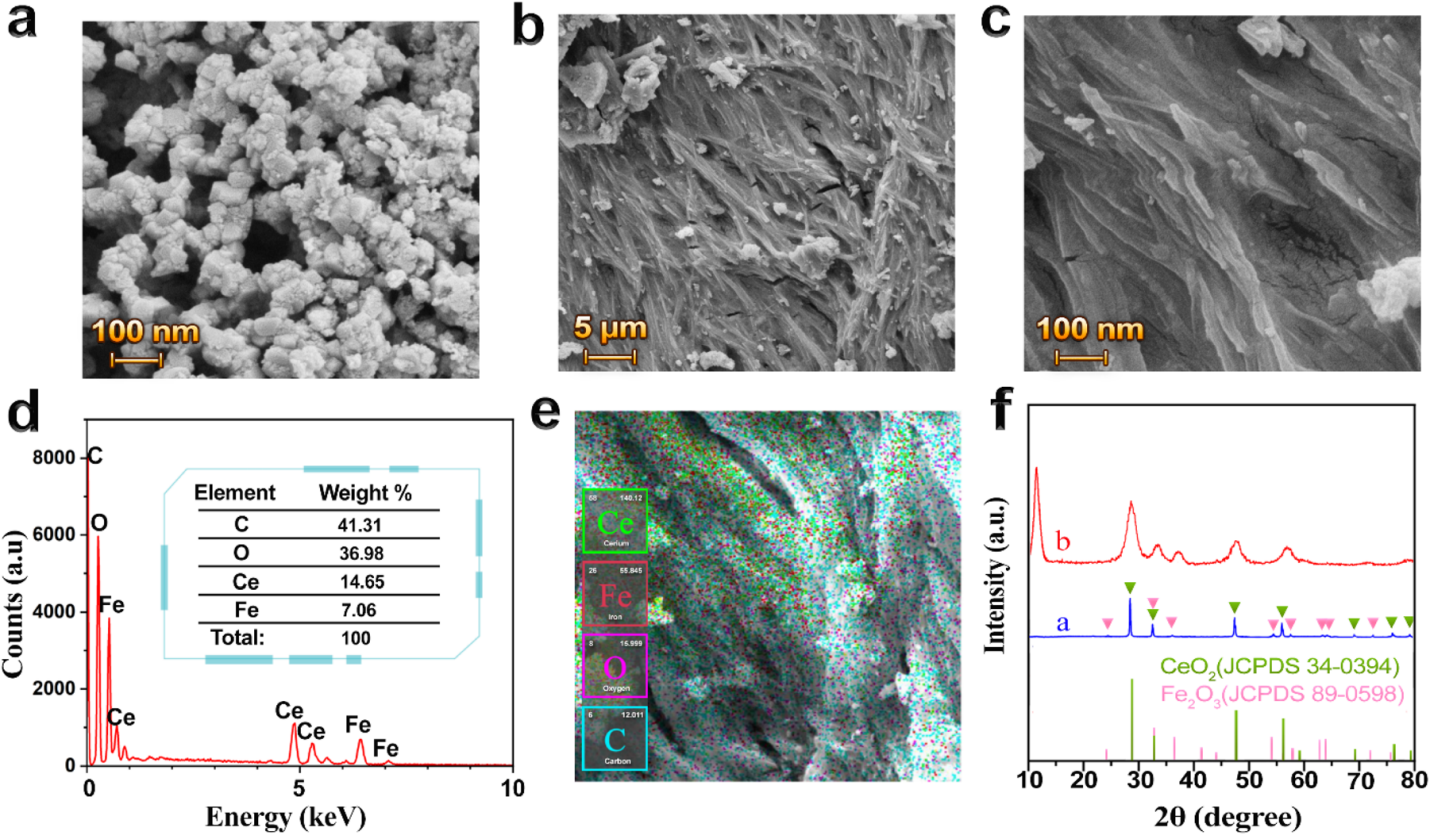
(a) FESEM image of nano-CeO_2_–Fe_2_O_3_. (b and c) FESEM image of nano-CeO_2_–Fe_2_O_3_–GO on the tag after curing at 180 °C. (d and e) EDS and EDS-MAP respectively, nono-CeO_2_–Fe_2_O_3_–GO after curing. (f) and (a) XRD of nano-CeO_2_–Fe_2_O_3_, the green triangles correspond to the diffraction pattern of the cubic CeO_2_ structure (JCPDS file no. 34-0394) and the pink triangles correspond to the hematite structure (JCPDS file no. 89-0598); (b) nano-CeO_2_–Fe_2_O_3_–GO after curing. The sharp peak at 12.1° is related to graphene oxide.

A nanocomposite material, consisting of a mixture of graphene oxide/cerium oxide and iron oxide (nano-CeO_2_–Fe_2_O_3_–GO), was developed and used as a sensing material (Fig. 1b). CeO_2_–Fe_2_O_3_ nanoparticles were produced using the methods mentioned in the “Experimental” section. The FESEM image of the prepared nanoparticles is presented in Fig. 4a, where the morphology and cubic structure of these nanoparticles are clearly visible.

The XRD analysis has confirmed the formation of nano-CeO_2_–Fe_2_O_3_, which is consistent with the XRD patterns of both CeO_2_ and Fe_2_O_3_, as illustrated in Fig. 4f. The pattern indicates that CeO_2_ is crystallized in a cubic structure (JCPDS file no. 34-0394), with six main reflections at 28.56° (111), 31.90° (200), 47.40° (220), 56.40° (311), 59.10° (222), 69.72° (400), 76.44° (331) and 78.54° (420). Additionally, Fe_2_O_3_ is present in the form of hematite and in a rhombohedral network structure, according to (JCPDS file no. 89-0598). Its diffraction peaks at 24.76° (012), 32.86° (104), 35.42° (110), 40.81° (113), 49.31° (024), 53.86° (116), 57.34° (122), 62.40° (214), 63.96° (300), 71.75° (1010), 75.22° (220).

In the second step, nano-CeO_2_–Fe_2_O_3_ was added to graphene oxide in varying ratios (1 to 10 mmol) to investigate the impact of nanoparticle loading on sensor performance. The amount of CeO_2_–Fe_2_O_3_ plays a critical role in determining the conductivity and sensitivity of the final sensing composite. Ethanol, AC, and AMP were then added to create a paste of nano-CeO_2_–Fe_2_O_3_–GO, which was cured using the UHP-GE paste method.

The EDS (energy dispersive X-ray spectroscopy) results depicted in Fig. 4d and e provide insight into the percentage of elements present in the nano-CeO_2_–Fe_2_O_3_ dispersed within the graphene oxide. These results also demonstrate a uniform dispersion of the elements throughout the sample. As shown in Fig. 4f(b), the characteristic diffraction peak at 12.0° confirms the presence of graphene oxide in the composite. Furthermore, the nano-CeO_2_–Fe_2_O_3_–GO patterns exhibit CeO_2_ peaks at 28.56° (111) and 47.40° (220), as well as rhomboid peaks at 32.86° (104) and 35.42° (110), indicating the presence of Fe_2_O_3_. However, the Fe_2_O_3_-related peaks are relatively weak and broad, likely due to the small crystallite size, lower relative content, and their fine dispersion within the GO matrix. Moreover, the post-curing step at 180 °C is not sufficient to induce significant grain growth, contributing to the observed broadening. Overall, these results demonstrate that AC can help to better bond graphene oxide with nanoparticles on different surfaces. The AC is decomposed by heat application and does not affect conductivity or resistance.

Due to the broad scope of this work, which included fabrication of the RFID tag in addition to the sensing layer, systematic variation of the Fe/Ce ratio was not performed. However, this parameter could significantly influence crystallinity and sensor performance and is therefore recommended as a topic for future research.

### 2.4 Preparation and properties of wireless hydrogen sensor

The sensors was prepared using the RFID tag with the aluminum substrate created in the previous step. The process involved covering the RFID tag with Kapton tape, leaving only a small portion of the antenna pattern (6 × 5 × 0.2 mm) exposed. Two gold electrodes were then carefully placed on either side of the cavity, as depicted in Fig. 1b. Next, the cavity was filled with nano-CeO_2_–Fe_2_O_3_–GO paste, and excess material was removed with silk. Finally, the tag was heated to 180 °C for 20 minutes to ensure optimal performance.

**Fig. 5:**
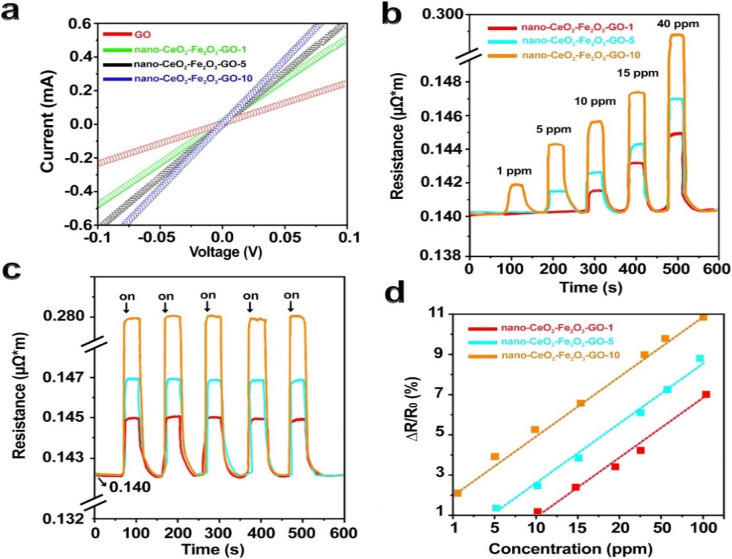
(a) The current–voltage curves of the sensors, which were fabricated using nano-CeO_2_–Fe_2_O_3_ on the surface of graphene oxide, were measured with a current value ranging from 5 to 10 amperes. (b) The resistance was observed to change as a function of hydrogen gas concentration over time. (c) The sensors were exposed to 40 ppm hydrogen intermittently and periodically. (d) The nono-CeO_2_–Fe_2_O_3_–GO sensor was calibrated for different concentrations of hydrogen gas.

To evaluate conductivity and nanoparticle distribution, *I*–*V* curves were recorded under ambient conditions. As shown in Fig. 5a, all samples exhibited linearity from −0.1 to 0.1 V, confirming ohmic contact behavior. Conductivity improved with increasing nano-CeO_2_–Fe_2_O_3_–GO content.

Sensor behavior was assessed by monitoring resistance changes in nano-CeO_2_–Fe_2_O_3_–GO-1, -5, and -10 tags under different H_2_ concentrations at 25 °C and 20% RH. Pure H_2_ gas was used in these tests (Fig. 5b). The resistance increased proportionally with H_2_ concentration, from 1 to 40 ppm. This trend is attributed to the interaction between adsorbed hydrogen and surface-adsorbed oxygen species, which modulate carrier density and enhance electron depletion effects in the semiconducting metal oxide nanoparticles.

Furthermore, nano-CeO_2_–Fe_2_O_3_–GO-1 failed to respond to H_2_ concentrations below 10 ppm, likely due to insufficient active surface area and limited adsorption capacity. In contrast, nano-CeO_2_–Fe_2_O_3_–GO-10 exhibited clear detection at 1 ppm, attributed to higher nanoparticle content enhancing surface reactions.

However, CeO_2_–Fe_2_O_3_–GO-10 also exhibited the highest baseline resistance, as observed in Fig. 5b. This is due to the semiconducting nature of CeO_2_ and Fe_2_O_3_ nanoparticles, which disrupt the conductive GO network when present in excess. Increased oxide content reduces percolation pathways and carrier mobility, resulting in higher overall resistance. This phenomenon has been similarly reported in oxide–graphene hybrid sensors in previous studies.^3,43^

To evaluate the dynamic response of nano-CeO_2_–Fe_2_O_3_–GOs and investigate their repeatability and stability, we exposed the sensors to H_2_ gas with a concentration of 40 ppm at room temperature (Fig. 5c), which demonstrated excellent stability and repeatability.

In addition, for calibration, the responses obtained from the sensors at different concentrations of H_2_ gas were recorded and processed (Fig. 5d). At low hydrogen concentrations, nonlinear behavior was observed, but when the hydrogen concentration increased, the behavior of the sensor became linear. Concentrations between 1 and 100 ppm show a linear behavior, which indicates the high sensitivity of the sensors to H_2_ gas.

This linearity confirms the sensor's effective response to hydrogen even in low ppm ranges and supports the practical utility of the CeO_2_–Fe_2_O_3_–GO-10 sample.

It was discovered that pristine graphene oxide does not respond to H_2_ gas due to the lack of an active surface to absorb H_2_ molecules. Furthermore, when AC remains undecomposed, the sensitivity to H_2_ gas is negligible due to the high resistance of the compound. However, after AC breakdown, the surface resistance decreases and the electrical conductivity improves.

The input impedance of the antenna terminal (*Z*_in_) can be expressed using Kirchhoff's law, as shown in eqn (1):1
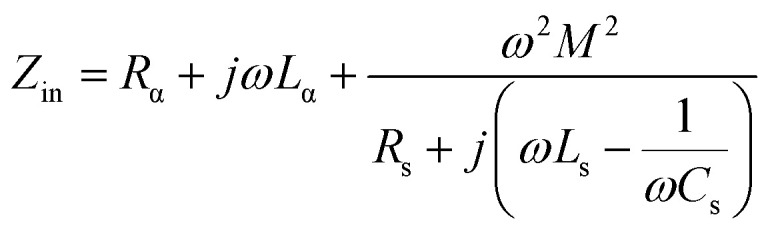


The mutual inductance between the sensor and antenna is denoted by “*M*”, while the angular frequency is represented by “*ω*”. The reflection value, *S*_11_ parameter, changes in response to H_2_ gas due to the resistance change of the variable resistor at the resonance frequency. Eqn (2) provides a simplified formula for *S*_11_, where *Z*_0_ is a fixed value of 50 Ω for all samples.2
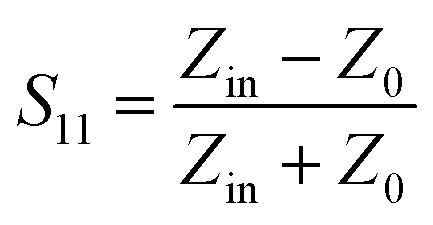


An increase in H_2_ concentration alters both the magnitude and phase of *Z*_in_, leading to a higher *S*_11_ value and a slight shift in the resonant frequency due to modified impedance matching.

**Fig. 6:**
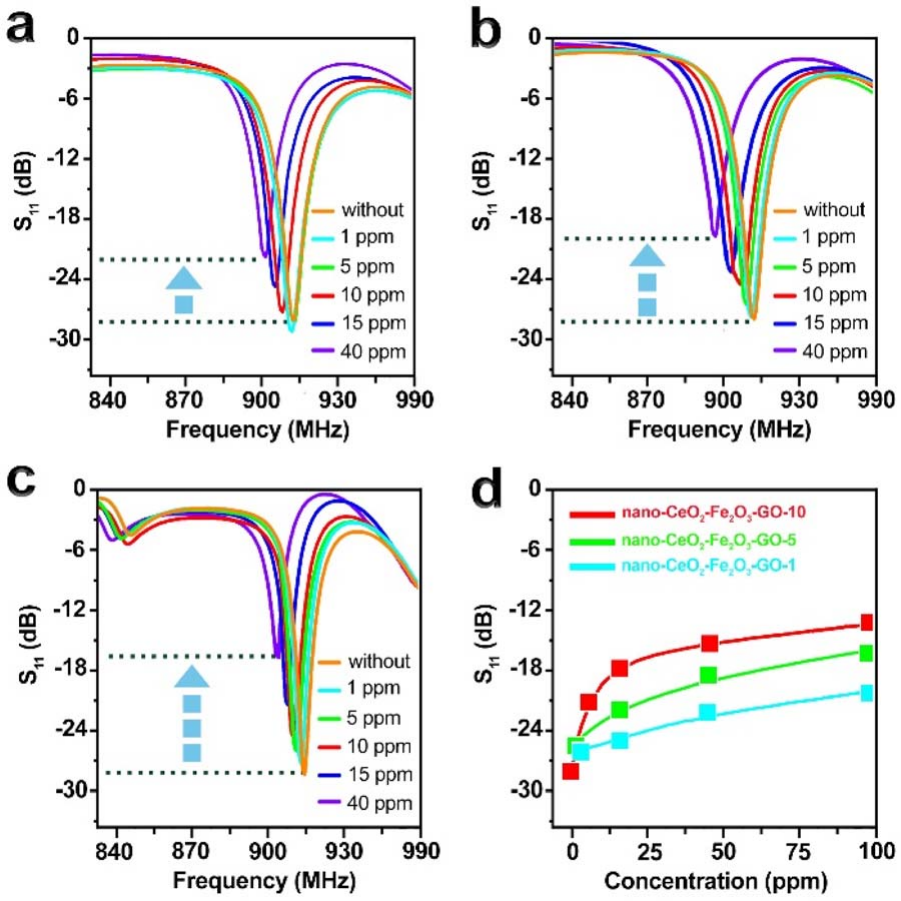
Changes in the reflectance of sensors in different concentrations of hydrogen gas for (a) nono-CeO_2_–Fe_2_O_3_–GO-1. (b) Nono-CeO_2_–Fe_2_O_3_–GO-5. (c) Nono-CeO_2_–Fe_2_O_3_–GO-10. (d) Calibration curves for prepared sensors as a function of H_2_ concentration, (red: nono-CeO_2_–Fe_2_O_3_–GO-10; green: nono-CeO_2_–Fe_2_O_3_–GO-5; cyan: nono-CeO_2_–Fe_2_O_3_–GO-1).

In Fig. 6, the observed gradual shift in the *S*_11_ curves with increasing H_2_ concentration is due to a change in the real and imaginary components of the sensor's input impedance (*Z*_a_). As the sensor's resistance increases (with more H_2_ adsorption), both the resonance depth and position of the minimum *S*_11_ value are affected. This results in a combined effect of magnitude reduction and slight frequency shift in the reflection profile. Such shifts are common in passive RFID sensors where the variable resistance alters the resonance condition of the tag antenna. Since the resonant frequency depends on both the real (*R*) and imaginary (reactive) parts of impedance, changes in *R*_s_ (sensor resistance) indirectly shift the resonance toward higher frequencies.^44,45^

To evaluate the response of the wireless hydrogen gas sensors, backscattering between an RFID reader and antennas connected to a network analyzer was employed. The analyzer generated frequency signals to activate the tag and collected impedance data within the desired range. Impedance matching between the antenna and the tag reflected the signal, allowing the analyzer to measure the reflection coefficient (*S*_11_). Variations in H_2_ concentration caused changes in the sensor's resistance, which modified the input impedance (*Z*_in) and led to observable shifts in the *S*_11_ signal, both in magnitude and frequency.

In the absence of H_2_, initial resistance varied based on the amount of nano-CeO_2_–Fe_2_O_3_–GO (GO-1, GO-5, GO-10), producing different *S*_11_ values. Among them, GO-10 showed the largest *S*_11_ shift, due to its higher nanoparticle content and lower resistance. Increasing H_2_ concentration gradually raised the circuit impedance, leading to changes in *S*_11_ while the resonance frequency remained nearly constant across all tags (Fig. 6a–c).

The alterations in *S*_11_ following a 10-second exposure to H_2_ were recorded and analyzed in relation to gas concentration. As illustrated in Fig. 6d, nano-CeO_2_–Fe_2_O_3_–GO-10 displayed the most significant *S*_11_ parameter shift across various concentrations. The CeO_2_–Fe_2_O_3_–GO nano-based sensors exhibit excellent response and recovery time (Fig. S6†), as well as favorable sensitivity when exposed to hydrogen gas after AC degradation. An increase in the ratio of nano-CeO_2_–Fe_2_O_3_ to graphene oxide results in an increase in sensitivity, with nano-CeO_2_–Fe_2_O_3_–GO-10 displaying exceptional sensitivity (1 ppm).

**Fig. 7:**
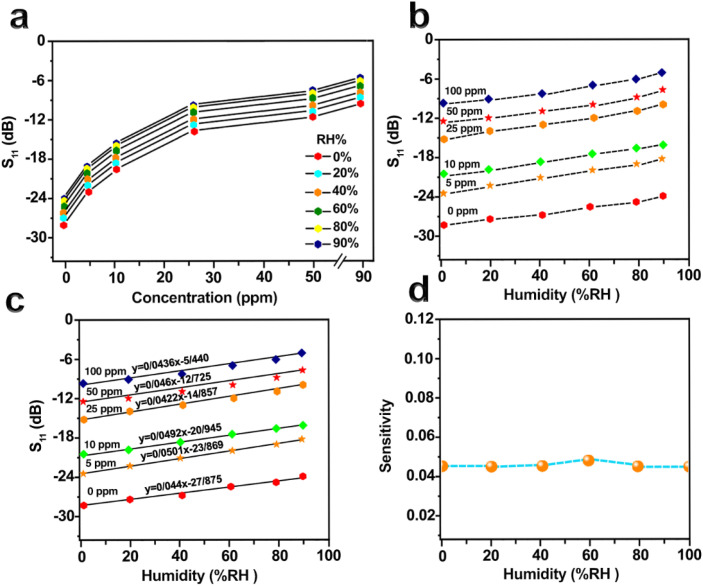
(a) The *S*_11_ were obtained from various H_2_ concentrations at relative humidity levels ranging from 0% to 90% RH. (b) The *S*_11_ were obtained at H_2_ concentrations ranging from 0 to 100 ppm under relative humidity levels of 0% to 90% RH. (c) Utilizing linear regression to fit the *S*_11_ values. (d) Employing a quartic polynomial curve to fit the average humidity sensitivity.

The environmental conditions under which hydrogen gas is measured are very important to obtain accurate and reliable results. Two key factors to consider are temperature and humidity. The ambient temperature was precisely controlled at 25 °C using a thermostat to ensure consistency throughout the tests. Secondly, the humidity level was also carefully controlled. Since humidity can interfere with gas adsorption mechanisms, changes in ambient moisture levels may significantly influence the sensor's response to H_2_. To investigate the impact of humidity, the sensor was exposed to various H_2_ concentrations (5, 10, 25, 50 and 100 ppm). Higher humidity levels reduce the surface adsorption of H_2_ molecules by competing for active sites, leading to a decrease in sensitivity and hence a reduced |*S*_11_| response, as shown in Fig. 7a. This observed reduction in |*S*_11_| response under high humidity is attributed to competitive adsorption: water molecules occupy active sites on the CeO_2_–Fe_2_O_3_–GO surface, thereby limiting H_2_ access and reducing the sensor's sensitivity. In practical scenarios, this can be mitigated by sensor surface modification or use of hydrophobic coatings to improve humidity tolerance.^46,47^

Fig. 7b further confirms a relatively linear decrease in |*S*_11_| values with increasing relative humidity. To determine the impact of humidity on the sensor's response at different H_2_ concentrations, we conducted a linear regression analysis on the collected data under varying humidity levels. The slope of the line was fitted and illustrated in Fig. 7c. Furthermore, we obtained diffusion coefficient curves of the sensor under different humidity levels, as displayed in Fig. 7d. By substituting the moisture content into the quartic polynomial fitting curve, we can compute the sensitivity coefficient.

The mechanism behind SMOX resistance sensing has been extensively studied and is quite intricate. According to prevalent theory, it involves a modification in surface electron depletion that results from the interaction between H_2_ and surface adsorbents.^48^

**Fig. 8:**
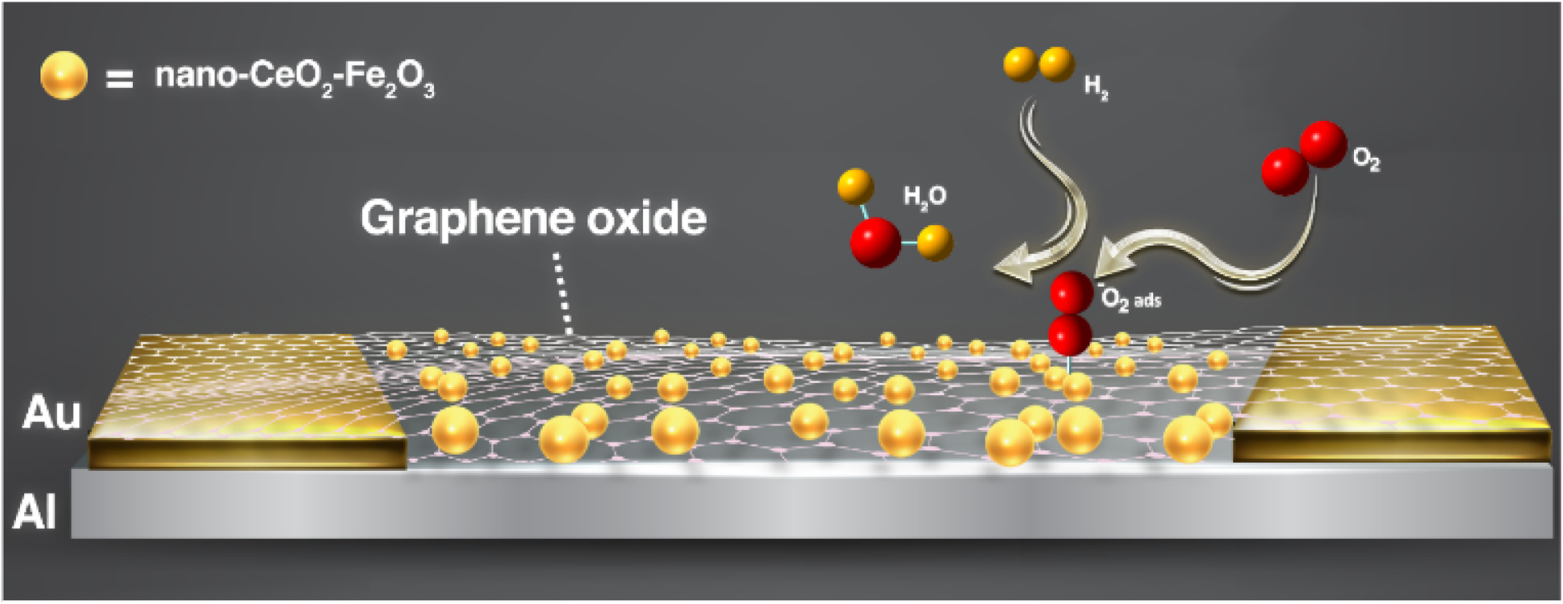
The hydrogen gas sensing mechanism of nono-CeO_2_–Fe_2_O_3_–GO is depicted in the schematic. When exposed to air, the sensor absorbs molecular oxygen and converts it into O_2_^−^. Upon exposure to hydrogen, the sensor facilitates the production of H_2_O, resulting in a decrease in resistance.

This sensing mechanism is not purely resistive; it involves catalytic redox reactions between H_2_ and chemisorbed oxygen species (O_2_^−^) on the surface of the sensing layer. In this composite, CeO_2_ provides oxygen storage and redox buffering *via* Ce^4+^/Ce^3+^ transitions, enhancing the response time and stability, while Fe_2_O_3_ contributes catalytic activity that accelerates H_2_ dissociation and reaction kinetics. When nano-CeO_2_–Fe_2_O_3_–GO is exposed to air, O_2_ molecules adhere to its surface and are transformed into O_2_^−^ on the surface of the material, as depicted in Fig. 8. This process leads to the formation of an electron layer near the surface, which increases resistance. However, when the surface of nano-CeO_2_–Fe_2_O_3_–GO is exposed to H_2_, an exothermic oxidation–reduction reaction takes place between H_2_ and O_2_^−^, which rapidly eliminates H_2_O molecules from the surface. This, in turn, reduces the resistivity of the nano-CeO_2_–Fe_2_O_3_–GOs. Upon returning the sensor to ambient air, the adsorbed species reabsorb the region, causing the resistance to revert to its initial level after the H_2_ response. Simply put, O_2_ adsorbs on the surface in air, increasing resistance; upon H_2_ exposure, it reacts with O_2_^−^, releasing electrons and lowering resistance. The process is reversible in air.

The mechanism of hydrogen measurement can be expressed as follows:

3 O_2_ (g) + e^−^ → O_2_^−^ (ads)

4 H_2_ (g) + 1/2O_2_^−^ (ads) → H_2_O + e^−^

when the sensor is integrated into the RFID tag, it initially behaves like a conventional passive tag in the absence of hydrogen gas, with stable impedance and standard *S*_11_ values. Upon exposure to H_2_, however, redox interactions at the CeO_2_–Fe_2_O_3_–GO surface increase the local resistance of the sensing region. This alters the input impedance of the antenna circuit, resulting in a measurable shift in the reflection coefficient (*S*_11_) while the resonant frequency remains largely unchanged. Thus, the system enables wireless, battery-free hydrogen detection by distinguishing the tag's response before and after gas exposure through impedance mismatch. This mechanism ensures minimal interference with the RFID communication protocol and maintains high tag performance.

A comparison of the sensors prepared in this work with other papers is given in Table 2.

**Table 2:** A comparison of the H_2_ sensors prepared in this work with other papers

Material	Temperature (°C)	Con.^a^ (ppm)	LOD^b^ (ppm)	*t*_res_ c/*t*_rec_^d^	Year/ref.
In_2_O_3_/CeO_2_	160	50	0.01	1/9	2018/43
Pd-NP/CeO_2_	350	10^6^	10	5/17	2021/49
CeO_2_/SnO_2_	300	60	5	17/24	2018/50
Pd@CeO_2_	350	100	1.4	60/360	2021/51
CeO_2_-Pd-PDA/rGO	100	6000	200	70/180	2022/52
Nano-CeO_2_–Fe_2_O_3_–GO	RT	40	1	8/60	This work

a Con = concentration.

b LOD = limit of detection.

c res = response time (second).

d rec = recovery time (second).

## 3. Experimental

### 3.1 Materials

Ethyl prop-2-enoate; methyl 2-methylprop-2-enoate; 2-methylprop-2-enoic acid (acrylate copolymer, *M*_w_ = 286.321, Henan Tianfu Chemical Co., Ltd), 2-amino-2-methyl-1-propanol (AMP), cerium nitrate hexahydrate (Ce(NO_3_)_3_·6H_2_O), ethanol (99%), ferric nitrate nonahydrate (Fe(NO_3_)_3_·9H_2_O), polyvinyl pyrrolidone (PVP, *M*_w_ = 58 000), graphene oxide and Hydrogen gas (Purity ≥99.99%) were purchased from Sigma-Aldrich. Ultra High Power (UHP) graphite electrodes (nipple) (Dan Carbon (Shanghai)).

### 3.2 Preparation of graphite paste

In order to create the graphite paste, the UHP graphite electrode (nipple) was first pulverized and sifted through a filter with a 1 micron mesh. The resulting powder was then combined with acrylate copolymer and 2-amino-2-methyl-1-propanol (AMP) in a mass ratio of 50 : 12 : 5, carefully selected to control electrical resistance and ensure proper adhesion. This mixture was then dispersed evenly in 100 ml of ethanol using a homogenizer for 30 minutes.

### 3.3 Fabrication of RFID tags

The antennas were simulated in ANSYS HFSS software and then made by screen printing. The UHP graphite paste prepared earlier was used to screen print on substrates of aluminum foil (15 microns thick), Kapton foil (15 microns thick), and fire-resistant paper (150 microns thick). After screen printing, the samples were cured in an oven for 20 minutes at 180 °C. the graphite layer that resulted from the process measured 25 microns in thickness. The weight of the remaining solid graphite layer was recorded at 52.54. To complete the experiment, the prepared tags were affixed to a soft PET foam measuring 75 mm × 20 mm × 0.2 mm. This foam boasts a dielectric constant (*ε*_r_) of 1.03. To complete the RFID tags, the packaged RFID chip (SL3S1213FTB0) was used. A silver-based conductive epoxy glue was used to connect the chip to the antenna and fill the gap of the UHF antennas.

### 3.4 Preparation of nano-CeO_2_–Fe_2_O_3_

In the preparation of nano-CeO_2_–Fe_2_O_3_, 5 grams of PVP was dissolved in 50 ml of ethanol. Then, 0.5 mmol of (Fe(NO_3_)_3_·9H_2_O) and 0.5 mmol of (Ce(NO_3_)_3_·6H_2_O) were added to it and stirred for 5 hours at 25 °C. The obtained solution was placed in an oven at 100 °C for 18 hours. The dry solid prepared in the previous step was then calcined for 4 hours at a temperature of 500 °C.

### 3.5 Preparation of nano-CeO_2_–Fe_2_O_3_–GO paste

To prepare the nano-CeO_2_–Fe_2_O_3_–GO paste, 20 grams of graphene oxide were added to 50 ml of absolute ethanol and stirred for 30 minutes. Next, 5 grams of acrylate copolymer and 2 ml of AMP were added and stirred for an hour at room temperature. The paste was then enriched with 1, 5 and 10 grams of nano-CeO_2_–Fe_2_O_3_, which had been meticulously prepared using the previous steps, and stirred for an additional 15 minutes. The resulting paste was then immediately ready for further applications.

### 3.6 Fabrication of RFID tag H_2_ sensor

For the preparation of the RFID tag H_2_ sensor, a Kapton tape was placed on the tag, covering its entire surface except for a hole measuring 6 mm × 5 mm × 0.2 mm. Two gold electrodes were placed in parallel with a gap of 70 micrometers on the surface of the RFID tag. The nano-CeO_2_–Fe_2_O_3_–GO paste prepared in the previous step was immediately screen-printed onto the cavity. Finally, it was placed at a temperature of 180 °C for 20 minutes.

### 3.7 Characterization and measurement

The compounds synthesized in this study were analyzed using PHILIPS X-ray diffraction (PW1730) to obtain their XRD patterns (Cu Kα_1_ radiation, *λ* = 1.54056 Å), while their Raman spectra were measured using a Teksan Raman spectrometer (TakRam N1-541). The morphology of the prepared samples was examined using a FESEM TESCAN field emission scanning electron microscope (MIRA III). The resistance and inductance of the samples were studied using a GW Instek LC meter (LCR-8201), and their sheet resistance was measured with a four-point probe (Jandel). To assess the effectiveness of the RFID tag antennas, we proceeded to connect them to the coaxial cable through an SMA (SubMiniature version A) connector. Then, utilizing a vector network analyzer (Agilent-8722 ET), we measured their *S*_11_ parameter (reflection coefficient) in the frequency spectrum of 800–1000 MHz. To read UHF RFID tags, a reader (KLM930) was used.

In order to assess the electrical characteristics and chemical resistance of the H_2_ sensor, we utilized RFID tag based on nano-CeO_2_–Fe_2_O_3_–GO. The tags were then tested in a sealed chamber (as depicted in Fig. S4†) at room temperature (25 °C). The RFID reader was positioned 15 cm above the tag in the chamber. Following this, the tags were stabilized within the chamber with fresh air flow for a period of two hours. To examine the efficacy of the tags, we exposed them to varying concentrations of H_2_ gas (ranging from 1–40 ppm), with careful control of gas flow *via* an MFC (FC-980 MFC Mass Flow Controller 50 SCCM Gas H_2_). Real-time monitoring of resistance was conducted by applying a constant current of 5–10 amps. After exposure to H_2_ gas for 5 minutes, free air was introduced into the vacuum chamber for a further 5 minutes. This step served to remove any hydrogen molecules attached to the CeO_2_–Fe_2_O_3_–GO nanoparticles, thereby restoring the active surface of the sensor and allowing it to be reused for repeated measurements of sensor performance. Each tag was read and checked individually. All measurements were made at 4 W EIRP output power.

The formula (*R* = Δ*R*/*R*_0_ × 100) was used to calculate the “Response” of the sensor to hydrogen gas (*R*). This formula measures the change in the resistance of the sensor (Δ*R*) compared to the initial resistance of the sensor (*R*_0_). In other words, the response of the sensor is the ratio of the change in resistance (Δ*R*) to the initial resistance (*R*_0_). This formula is commonly employed to gauge a sensor's response to a specific gas.^19^ All tests are conducted at room temperature (25 °C). Following this, the sensors are stabilized in air for two hours before being tested to check their functionality. Pure hydrogen gas is injected into the chamber for 5 minutes, followed by free air for the next 5 minutes. Throughout the investigation, the sensors are exposed to varying concentrations of H_2_ gas, ranging from 1–40 ppm. To clarify, we measured the initial resistance of the sensor (*R*_0_) in clean air (without H_2_ gas). When H_2_ gas was injected into the sensor, the resistance visibly changed, denoted as Δ*R*. For example, the initial resistance of the sensor (*R*_0_) was 0.14 µΩ m (at room temperature and 20% RH). In the presence of 1 ppm of hydrogen gas (in the nano-CeO_2_–Fe_2_O_3_–GO-10), the resistance of the sensor reached 0.14252 µΩ m (Δ*R* = 0.00252 µΩ m). Using formula (*R* = Δ*R*/*R*_0_ × 100), we calculated the response of the sensor to hydrogen gas as follows: *R* = (0.00252 µΩ m)/(0.14 µΩ m) ×100 = 1.8. Therefore, the response of the sensor to hydrogen gas in this example is equal to 1.8 (Fig. S5a†). The sensitivity of the prepared sensors was determined by calculating the response graph in relation to the concentration, as shown in Fig. S5c and d.† Additionally, Fig. S5a† presents the real-time dynamic response of the sensor under repeated hydrogen exposure and recovery cycles, confirming its wireless operation and reversibility.

## 4. Conclusion

In this study, RFID tags were successfully fabricated using a low-cost and high-conductivity graphite paste derived from UHP graphite electrodes, combined with additives such as acrylate copolymer (AC), AMP, and ethanol. After curing at 180 °C, the tags exhibited low resistivity (as low as 0.14 µΩ m on aluminum substrates), demonstrating that the decomposition of AC improved electrical conductivity. These tags also offered long read ranges-up to 10 m on metallic and 6 m on non-metallic substrates—highlighting their potential as sustainable alternatives to traditional metal or graphene-based antennas. Furthermore, an RFID-based wireless hydrogen sensor was developed by incorporating nano-CeO_2_–Fe_2_O_3_–graphene oxide composites into the tag structure. The sensor demonstrated excellent sensitivity, detecting H_2_ concentrations as low as 1 ppm without the need for an external power source. The system maintained stable performance in a passive RFID configuration, making it suitable for low-power, remote gas monitoring applications. Overall, the presented technology combines the advantages of printable graphite antennas and redox-active sensing materials, offering a promising platform for next-generation RFID-based environmental and industrial monitoring systems.

## Data availability

All data generated or analyzed during this study are included in this published article and its ESI files.†

## Conflicts of interest

There are no conflicts to declare.

## Supplementary Material

RA-015-D5RA00587F-s001
