# Supplementary material for: High conductivity graphite paste for radio frequency identification tag with wireless hydrogen sensor based on CeO2–Fe2O3–graphene oxide
Source: RSC Adv. 2025 Apr 22;15(16):12773–84. doi: 10.1039/d5ra00587f (PMC12013617; doi:10.1039/d5ra00587f)
Supplement: RA-015-D5RA00587F-s001 [file RA-015-D5RA00587F-s001.pdf]

Supplementary Information (SI) for RSC Advances.
This journal is © The Royal Society of Chemistry 2025

Supporting Information

High conductivity graphite paste for radio frequency identification tag with wireless hydrogen sensor based on CeO2–Fe2O3–graphene oxide.

Hossein Mojtabazadeh [a] and Javad Safaei-Ghomi [a,*]

[a]*Department of Organic Chemistry, Faculty of Chemistry, University of Kashan, Kashan, I. R. Iran*

1- Optimization of UHP-GE pastes on different substrates

* Corresponding author. Department of Organic Chemistry, Faculty of Chemistry, University of Kashan, Kashan, P.O. Box 87317-51167, I. R. Iran. Email: safaei@kashanu.ac.ir. Phone: +98-31-55912385.

Table S1. Comparing the resistance of UHP-GE pastes with different substrates at different curing temperatures.

Paste Type	Temperature	Curing Time (min)	Resistance (μΩ*m)
UHP-GE@ Al[a]	80°C	20	~10^8
	100°C	20	~5
	120°C	20	1.5
	140°C	20	0.8
	160°C	20	0.15
	180°C	20	0.14
	200°C	20	0.14
	220°C	20	0.14
UHP-GE@ Kapton foil	80°C	20	~10^9
	100°C	20	~1000
	120°C	20	2.4
	140°C	20	2.2
	160°C	20	2.1
	180°C	20	2.1
	200°C	20	2.1
	220°C	20	2.1
UHP-GE@ fire-resistant paper	80°C	20	~10^9
	100°C	20	~1000
	120°C	20	2.5
	140°C	20	2.2
	160°C	20	2.1
	180°C	20	2.1
	200°C	20	2.1
	220°C	20	2.1

[a]Al = Aluminium.

2- EDS of UHP-GE and UHP-GE paste

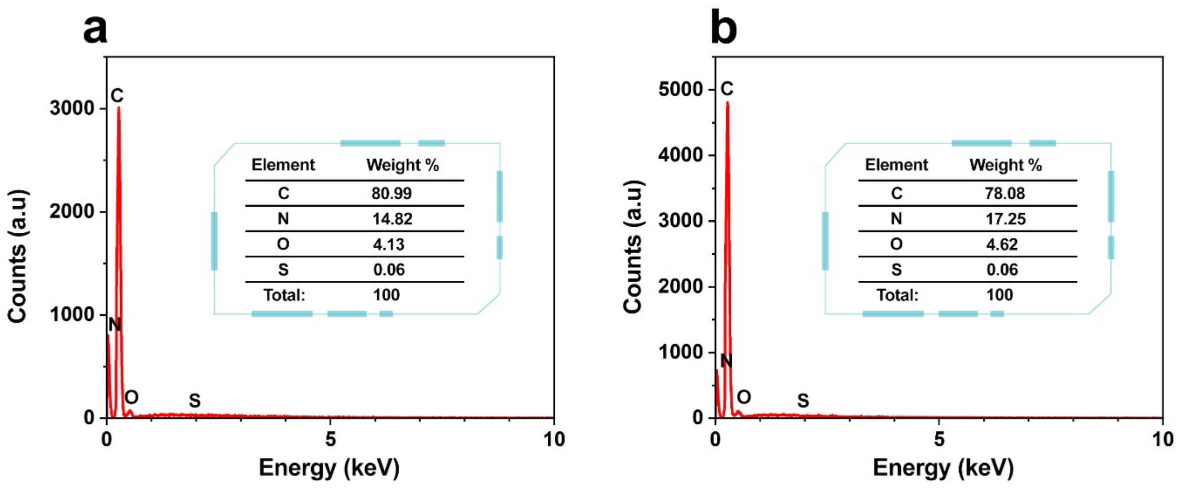

Figure S1: Effects of treatment on graphite powder. EDS spectrum of UHP-GE powder before (a) and after (b) treatment.

3- EDS mapping of UHP-GE and UHP-GE paste

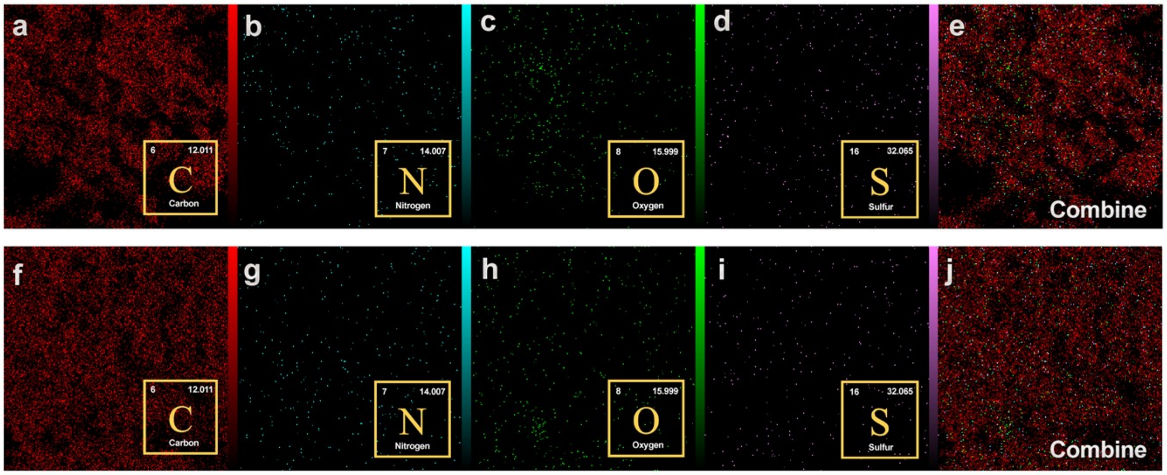

Figure S2: Effects of treatment on UHP-GE powder.

a-e) UHP-GE powder before treatment and (f-j) after treatment.

4- Optimizing the designed antenna

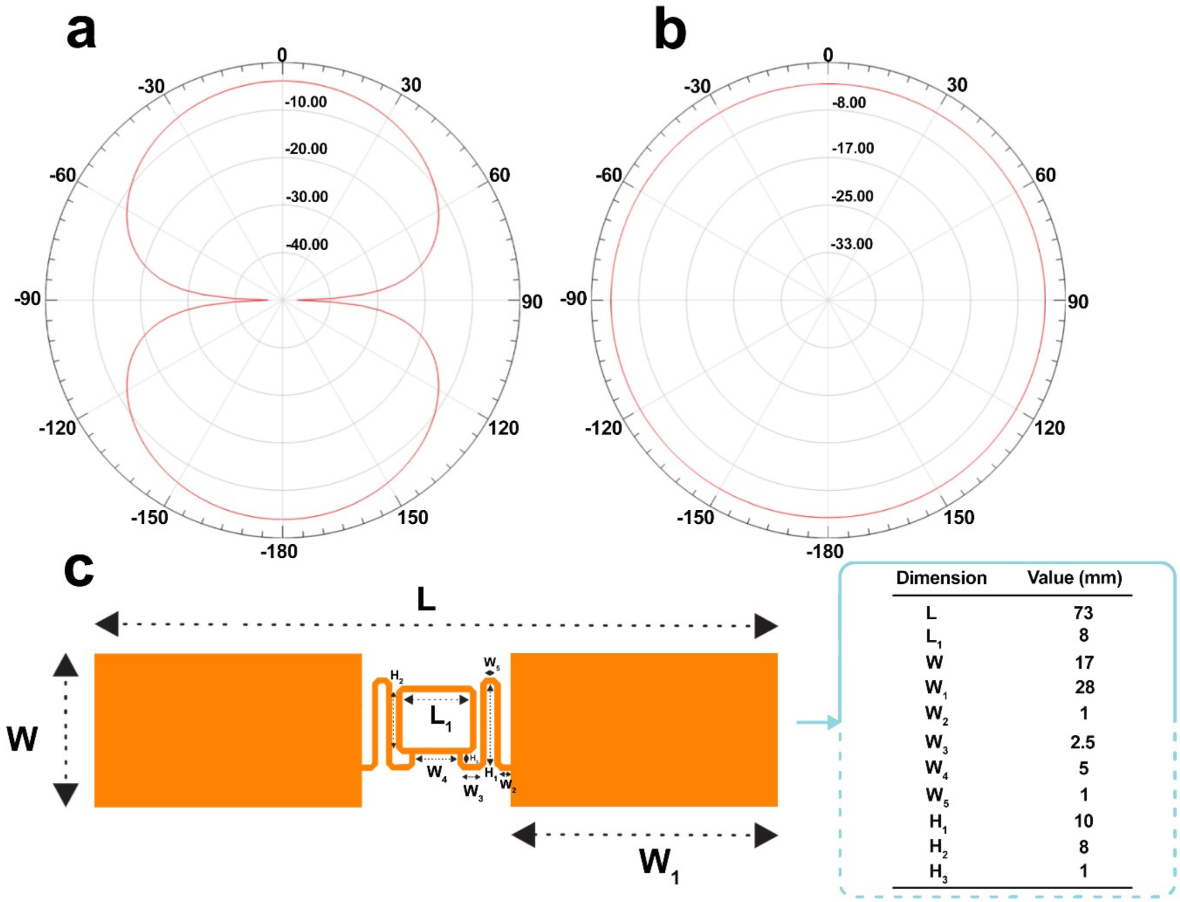

Figure S3: a) The H-plane and b) E-plane radiation patterns of the antenna model were simulated using HFSS software. c) Structure diagram.

5- RFID tags antenna application

Table S2. Important parameters of the designed dipole antenna.

type	VSWR	reflection coefficient (S_{11})	Transmitted Power	Reflected Power
UHP-GE@ Al	1.08	-28.299	99.851	0.148
UHP-GE@ FRP[a] or UHP-GE@ KF[b]	1.58	-12.964	94.946	5.053

[a]FRP=fire-resistant paper, [b]KF=kapton foil.

6- Characteristics of the UHF RFID tags

Table S3. Basic features of UHF RFID tags produced.

	Tag a	Tag b	Tag c	Tag d
Conductive material	UHP-GE	UHP-GE	UHP-GE	aluminum
Substrate	kapton foil	fire-resistant paper	aluminum foil	kapton foil
Rs (Ω sq^{-1})[a]	0.08	0.08	0.01	0.00289
σ(S m^{-1})[b]	4.75×10^{5}	4.75×10^{5}	4×10^{6}	1.38×10^{7}
S_{11}	-13	-13	-28	-37
Read range (m)	6	5.9	10	15.2

[a] Rs=sheet resistance, [b]σ=conductivity

7- The environmental conditions under which H_2

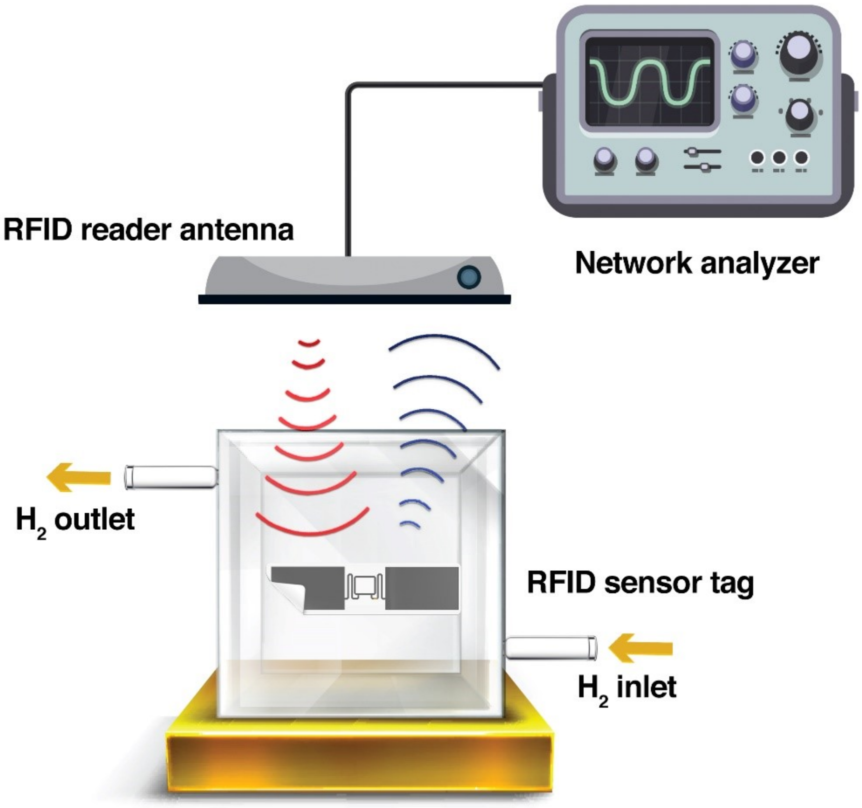

Figure S4: illustrates a wireless system designed for measuring H_2 gas, which includes an RFID reader, a network analyzer, and a sealed container that houses an RFID tag. The tag is placed 15 cm away from the antenna, and all tests are conducted at room temperature (25°C). Following this, the labels are stabilized in air for two hours before being subjected to a test to check the sensor's functionality. Pure hydrogen gas is injected into the chamber for 5 minutes, followed by free air for the next 5 minutes. Throughout the investigation, the tags are exposed to varying concentrations of H_2 gas, ranging from 1-40 ppm.

8- Sensitivity of sensors in different concentrations of H_2 gas

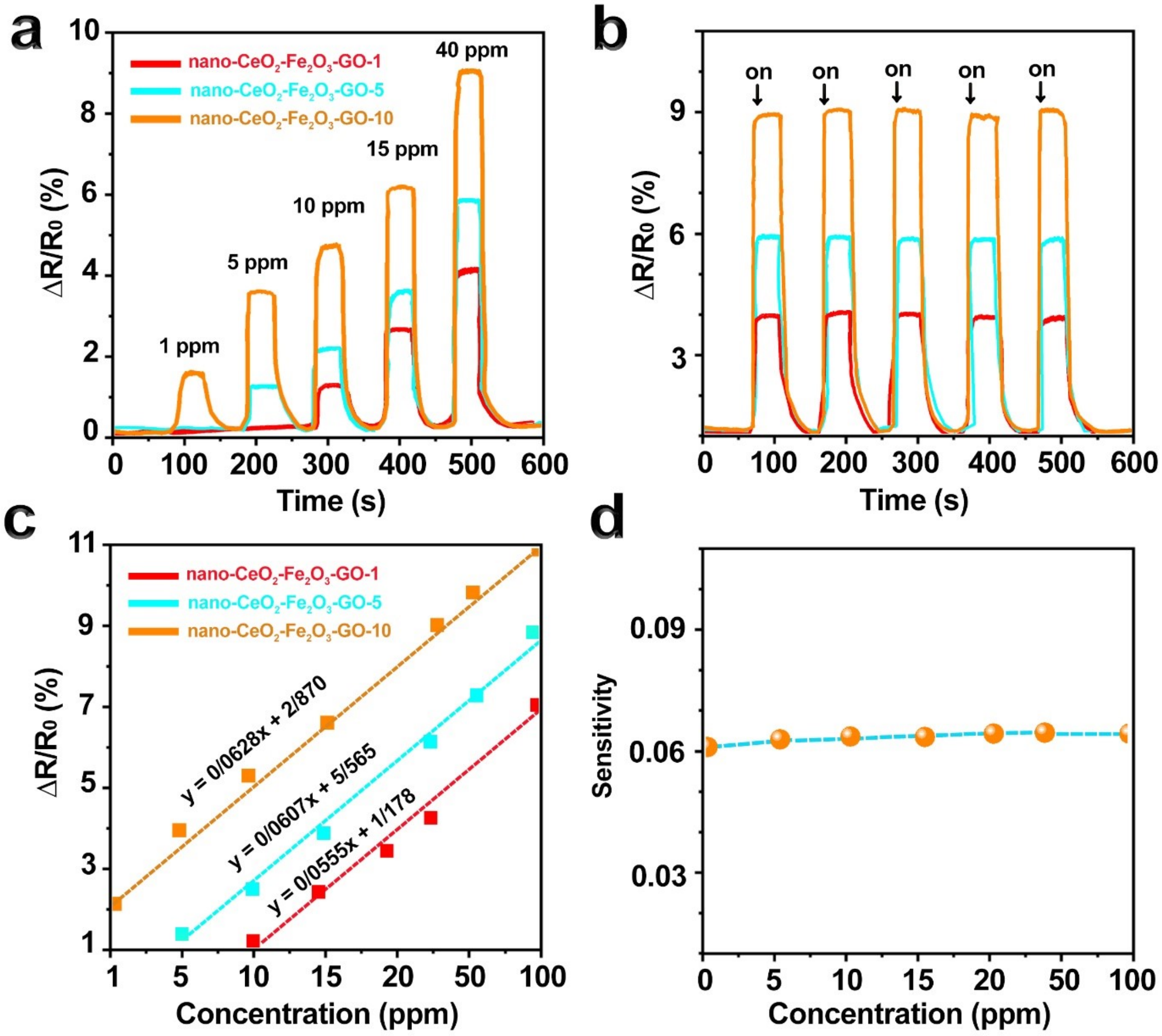

Figure S5: a) The sensor's response was measured at different times and concentrations. b) The sensors were exposed to 40 ppm hydrogen gas periodically. c) The sensor's response was plotted against the hydrogen gas concentration. d) The sensitivity to hydrogen gas was fitted using a quartic polynomial curve.

9- Response and recovery time of hydrogen detection sensors

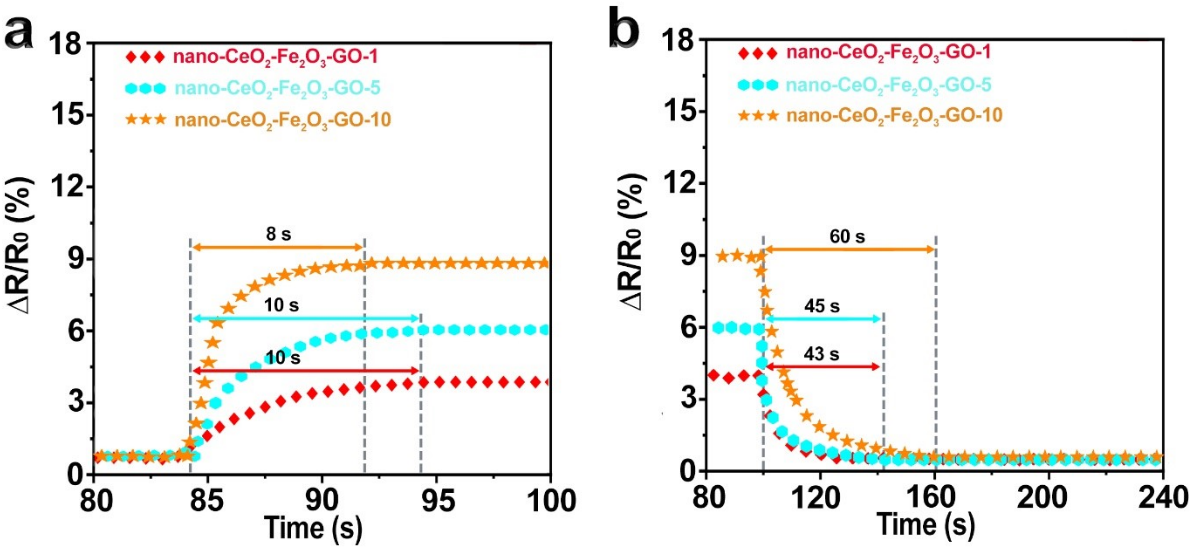

Figure S6: a) Response time and b) Recovery time of sensors based on nano-CeO_2-Fe_2O_3-GO in 40 ppm concentration of H_2 gas.
